# Role of the DNA Base Excision Repair Protein, APE1 in Cisplatin, Oxaliplatin, or Carboplatin Induced Sensory Neuropathy

**DOI:** 10.1371/journal.pone.0106485

**Published:** 2014-09-04

**Authors:** Mark R. Kelley, Yanlin Jiang, Chunlu Guo, April Reed, Hongdi Meng, Michael R. Vasko

**Affiliations:** 1 Department of Pediatrics, Herman B Wells Center for Pediatric Research, Indianapolis, Indiana, United States of America; 2 Department of Pharmacology & Toxicology, Indiana University School of Medicine, Indianapolis, Indiana, United States of America; Indiana University School of Medicine, United States of America

## Abstract

Although chemotherapy-induced peripheral neuropathy (CIPN) is a dose-limiting side effect of platinum drugs, the mechanisms of this toxicity remain unknown. Previous work in our laboratory suggests that cisplatin-induced CIPN is secondary to DNA damage which is susceptible to base excision repair (BER). To further examine this hypothesis, we studied the effects of cisplatin, oxaliplatin, and carboplatin on cell survival, DNA damage, ROS production, and functional endpoints in rat sensory neurons in culture in the absence or presence of reduced expression of the BER protein AP endonuclease/redox factor-1 (APE1). Using an *in situ* model of peptidergic sensory neuron function, we examined the effects of the platinum drugs on hind limb capsaicin-evoked vasodilatation. Exposing sensory neurons in culture to the three platinum drugs caused a concentration-dependent increase in apoptosis and cell death, although the concentrations of carboplatin were 10 fold higher than cisplatin. As previously observed with cisplatin, oxaliplatin and carboplatin also increased DNA damage as indicated by an increase in phospho-H2AX and reduced the capsaicin-evoked release of CGRP from neuronal cultures. Both cisplatin and oxaliplatin increased the production of ROS as well as 8-oxoguanine DNA adduct levels, whereas carboplatin did not. Reducing levels of APE1 in neuronal cultures augmented the cisplatin and oxaliplatin induced toxicity, but did not alter the effects of carboplatin. Using an *in vivo* model, systemic injection of cisplatin (3 mg/kg), oxaliplatin (3 mg/kg), or carboplatin (30 mg/kg) once a week for three weeks caused a decrease in capsaicin-evoked vasodilatation, which was delayed in onset. The effects of cisplatin on capsaicin-evoked vasodilatation were attenuated by chronic administration of E3330, a redox inhibitor of APE1 that serendipitously enhances APE1 DNA repair activity in sensory neurons. These outcomes support the importance of the BER pathway, and particularly APE1, in sensory neuropathy caused by cisplatin and oxaliplatin, but not carboplatin and suggest that augmenting DNA repair could be a therapeutic target for CIPN.

## Introduction

Cisplatin, oxaliplatin, and carboplatin, are widely used as primary therapies in various types of tumors, including but not limited to testicular, bladder, ovarian, lung, esophagus, stomach, and colon cancer [1]–[5]. A major limitation with the use of platinum drugs is the peripheral neuropathy that occurs in a significant number of patients. With cisplatin, the neuropathy develops during ongoing therapy in approximately 50% of patients and its severity is, in part, dependent on the total amounts of drug that the patient receives [4], [6]–[8]. Furthermore, in a significant number of patients, the neuropathy can persist long after therapy is discontinued and in some cases is irreversible [4], [7]–[9]. Chronic administration of oxaliplatin also can result in peripheral neuropathy that occurs during chronic therapy and is similar in symptom presentation, in frequency, and in duration to cisplatin-induced neurotoxicity [10]–[12]. In contrast to cisplatin, however, acute administration of oxaliplatin in a large percent of patients receiving the drug also causes an acute and reversible neurotoxicity characterized by pain, focus weakness, and increased sensitivity to cold [4], [7], [12]–[14]. High concentrations of carboplatin have been reported to cause peripheral neuropathy in patients receiving multiple drug therapy [15]. Despite this, most studies suggest that the incidence of neuropathy after chronic carboplatin therapy is less frequent and less severe than that observed with cisplatin or oxaliplatin [16], [17].

Although the mechanisms by which platinum drugs produce peripheral neuropathy remain unknown, evidence supports the notion that the neurotoxicity is secondary to DNA damage in sensory neurons. Platinum-induced cytotoxicity involves formation of intrastrand and interstrand adducts in DNA [18], and accumulation of these adducts in rat sensory neurons correlates with damage in sensory neurons [19], [20]. Chronic administration of cisplatin or oxaliplatin to rodents or long-term exposure of isolated sensory neurons damages sensory neurons and causes apoptosis, depending on the doses/concentrations used [21]–[26]. Furthermore, cisplatin-induced neurotoxicity is exacerbated in mice deficient in nucleotide excision repair (NER), compared to mice with NER intact [27]. Like cisplatin and oxaliplatin, administering carboplatin to rats also produces toxicity in sensory neurons and accumulation of the drug in the dorsal root ganglia [28]. Given that these drugs result in formation of platinum adducts, but exhibit differences in incidence and types of neurotoxicity in patients, the question remains whether the neuropathy is secondary to formation of adducts in sensory neurons or is mediated by other actions.

Recent studies suggest that cisplatin-induced toxicity also may be mediated by the ability of the platinating agent to increase formation of reactive oxygen species (ROS) [29]–[33]. An increase in ROS could result in oxidative DNA damage, which could also contribute to the neurotoxicity. The base excision repair (BER) pathway is the major pathway for correctly repairing oxidative DNA induced damage [34]–[39]. Within this pathway, apurinic/apyrimidinic endonuclease/redox effector factor (APE1) is a critical enzyme that is essential for repair by cutting the DNA backbone at baseless sites (abasic) in DNA after the removal of the damage base [38], [40]–[42]. Our previous published work demonstrated that augmenting the DNA repair function of APE1 significantly decreased cisplatin-induced toxicity in sensory neurons [7], [43], [44]. However, the role of APE1 on carboplatin- and oxaliplatin-induced neurotoxicity has not been studied, nor has the use of a small molecule to enhance APE1 DNA repair activity and neuronal cell protection. Consequently, we examined the effects of the platinum compounds on cell survival and function of sensory neurons in culture in the absence or presence of reduced expression of APE1 and whether these agents produce significant ROS. We also examined the difference in the effects of platinum agents on alterations in vasodilation induced by activation of sensory nerve endings in the rat hindpaw as a translational model for neuropathy.

We demonstrate that the loss of APE1 function increases sensitivity of sensory neuronal cultures to cisplatin and oxaliplatin, but not to carboplatin. This relationship correlates with the level of general ROS and DNA damage produced by the platinum agents, as well as a marker for specific oxidative DNA base damage: i.e. 8-oxoguanine. We also observe differences in the platinum agents on peripheral blood flow and demonstrate the *in vivo* use of a small molecule, which shows protective activity against cisplatin-induced neuropathy. These findings are particularly noteworthy in light of the recent report by the American Society of Clinical Oncology (ASCO) which determined there are no current clinical agents recommended for the prevention of CIPN [45].

## Results

### Reducing APE1 expression in sensory neuronal cultures increases cisplatin and oxaliplatin, but not carboplatin- induced cell loss

**Figure 1:**
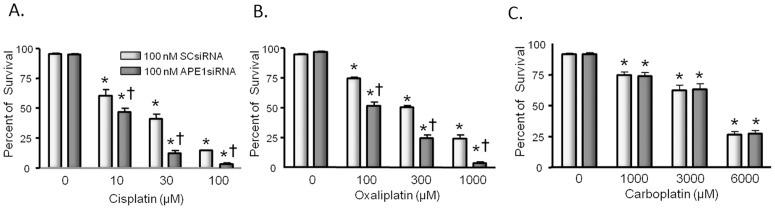
Reducing the expression of APE1 augments the ability of cisplatin and oxaliplatin, but not carboplatin to reduce cell viability in sensory neuronal cultures. Neuronal cultures were treated with siRNAs on days 3–5 in culture then exposed to various concentrations of platins for 72 hours starting on day nine in culture. Cell viability as measured by trypan blue exclusion was determined on day 12 in culture from three independent harvests. Each column represents the mean ± SEM of percent survival of cells from cultures treated with scrambled siRNA (SCsiRNA; lightly shaded columns) or with APE1si RNA (heavy shaded columns), then exposed to various concentrations of cisplatin (panel A), oxaliplatin (panel B), or carboplatin (panel C) as indicated. An asterisk indicates significant difference in survival in the absence or presence of drug treatment, whereas a cross indicates significant difference in cultures treated with SCsiRNA versus APE1siRNA using ANOVA and Tukey's post hoc test.

We have previously demonstrated that reducing the expression of APE1 results in increased cell death in sensory neuronal cultures after treatment with cisplatin and this correlates with an increase in ROS production [24]. To determine whether this is a global phenomenon with the other platinating agents, neuronal cultures were exposed to APE1 siRNA or scramble siRNA (SCsiRNA) on days 3–5 in culture, then exposed to various concentrations of platins for 72 hours starting on day 9 in culture. Cell viability as measured by trypan blue exclusion was determined on day 12 in culture from 3 independent harvests. In these and subsequent experiments, exposing cultures to APE1siRNA resulted in a significant reduction in APE1 expression and endonuclease activity (see Figure S1). In the combined studies using cisplatin, oxaliplatin, or carboplatin, APE expression after APE1 siRNA was reduced to 18 ± 4%, 11 ± 3% and 11 ± 5%, respectively. As we observed in our previous studies, cisplatin exposure resulted in a concentration-dependent decrease in cell viability that was significantly greater with reduced expression of APE1 (Figure 1A). A significant loss of viability was observed with 10 µM cisplatin, and there was a maximal loss in cells treated with APEsiRNA. Treating neuronal cultures with oxaliplatin also reduced viability in a concentration-dependent manner, and this effect was significantly higher when APE1 expression was reduced by APE1siRNA (Figure 1B). With oxaliplatin, significant loss in viability was seen with 100 µM, and the maximal cell loss after APE1 knockdown was seen with 1000 µM. Carboplatin treatment also significantly reduced cell viability but at higher concentrations than either cisplatin or oxaliplatin. In contrast to the other platinating agents, reducing APE1 expression did not have any significant effect on carboplatin-induced cell death (Figure 1C).

**Figure 2:**
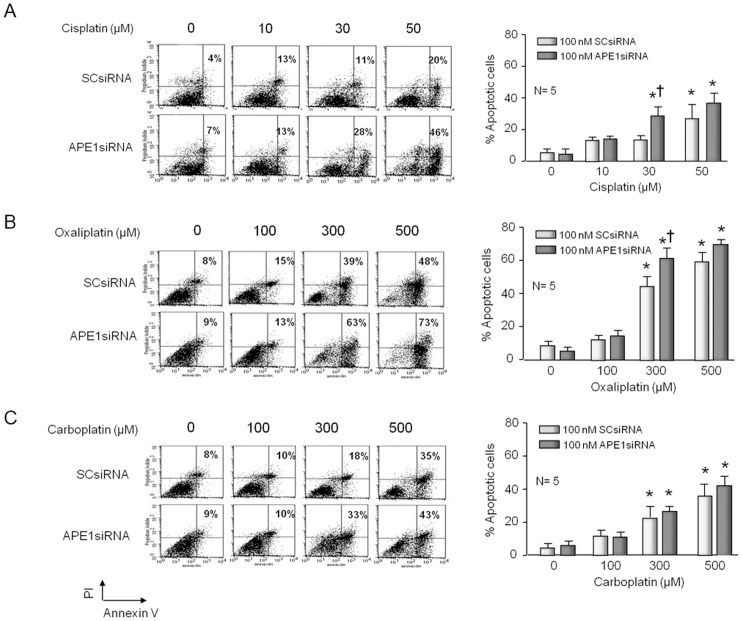
Apoptosis induced by cisplatin and oxaliplatin, but not carboplatin, is increased by reducing the expression of APE1 in sensory neuronal cultures. Neuronal cultures were treated with siRNAs on days 3–5 in culture then exposed to various concentrations of platins for 72 hours starting on day nine in culture. Cell apoptosis was detected by Annexin-V and PI staining and FACS analyses after cells were grown for 12 days. The left panels show representative fluorescence-activated cell sorting (FACS) for cells treated with various concentrations of cisplatin (A), oxaliplatin (B), or carboplatin (C) and scrambled siRNA (SCsiRNA) or APE1siRNA as indicated. The panels on the right show the quantification of data from five independent harvests. Each column represents the mean ± SEM of the percent of apoptotic cells from cultures treated with SCsiRNA (lightly shaded) or with APE1siRNA (heavy shaded) and various concentrations of cisplatin (A); oxaliplatin (B) or carboplatin (C) as indicated. An asterisk indicates significant difference in survival in the absence or presence of drug treatment, whereas a cross indicates significant difference in cultures treated with SCsiRNA versus APE1siRNA using Student's *t*-test.

Platinum agents bind to DNA forming a variety of platinum-DNA adducts [46] as well as increasing ROS production with can cause oxidative DNA damage [47].These actions can lead to an increase in cell death which is usually driven by apoptosis. Consequently, we quantified the level of apoptosis induced by platinating agents in sensory neuronal cultures. Cultures were exposed to APEsiRNA on days 3–5 in culture then exposed to various concentrations of platins for 72 hours starting on day 9 in culture. Annexin-V and PI staining detected cell apoptosis and FACS analyses after cells were grown for 12 days. As observed with trypan blue exclusion, treating neuronal cultures with the platins for 72 hours resulted in a concentration-dependent increase in apoptosis (Figure 2). With cisplatin, apoptosis was observed after exposure 30 or 50 µM, whereas with oxaliplatin and carboplatin apoptosis was observed at 10-fold higher concentrations. The platin-induced apoptosis was observed in cultures pretreated with SCsiRNA or APE1siRNA. However, the reduction of APE1 expression after APE1siRNA resulted in a further increase in the percent of apoptotic cells in both cisplatin and oxaliplatin treated cells (Figure 2A and 2B), but not in carboplatin treated cells (Figure 2C); which is consistent with the trypan blue exclusion studies in Figure 1.

### APE1 knockdown increases DNA damage induced by oxaliplatin, but not by carboplatin in sensory neuronal cultures

**Figure 3:**
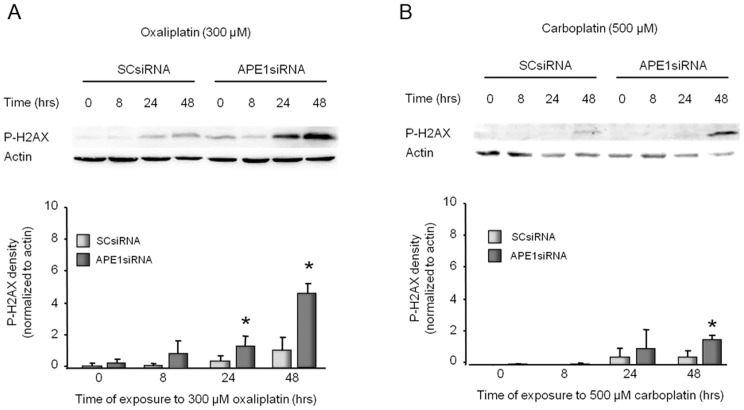
Platinum-induced phosphorylation of H2AX in sensory neuronal cultures is increased by reducing APE1 expression. The top panels show representative Western blots of phospho-H2AX (P-H2AX) and actin from cultures prior to and after 8, 24 and 48 hours of exposure to 300 µM oxaliplatin (A) or 500 µM carboplatin (B). Cultures were exposed to SCsiRNA or APE1siRNA as indicated. The bottom panels represent the densitometry of P-H2AX expression normalized to actin from three independent experiments. The columns represent the mean ± SEM from cultures treated with SCsiRNA (lightly shaded columns) or APE1siRNA (heavy shaded columns) prior to or after exposure to 300 µM oxaliplatin (A) or 500 µM carboplatin (B). An asterisk indicates a statistically significant increase in P-H2AX density in cells treated with APE1siRNA compared to those treated with SCsiRNA. Cisplatin data can be found in our previous publication [24].

To determine whether exposure to platinating agents produces DNA double-strand breaks, we measured the ability of oxaliplatin or carboplatin to augment that amount of phosphor-H2AX (P-H2AX) in the absence or presence of reduced APE1 expression. For these studies, cultures were transfected with SCsiRNA or APE1siRNA on days 3–5 in culture then exposed to 300 µM oxaliplatin or 500 µM carboplatin. The amount of P-H2AX was determined by Western blotting on day 12 after 0, 8, 24, or 48 hours of exposure to the platins. When cultures treated with SCsiRNA are exposed to platins for 8 or 24 hours, there is no significant increase in P-H2AX compared to controls (Figure 3). After 48 hours, however, there is a small increase in P-H2AX. When cells are pretreated with APE1siRNA, there is a significant increase in P-H2AX after 24 hours of exposure to oxaliplatin (2-fold) and 48 hours of exposure to oxaliplatin (10-fold) or carboplatin (2-fold) (Figure 3). The level of P-H2AX was higher in oxaliplatin treated cells (Figure 3A) than those treated with carboplatin (Figure 3B). In addition, carboplatin did not show as much of an increase in P-H2AX following APE1 reduction (2-fold at 48 hours) when compared to cells treated with oxaliplatin (5-fold). Similar studies with cisplatin have previously been published [24].

**Figure 4:**
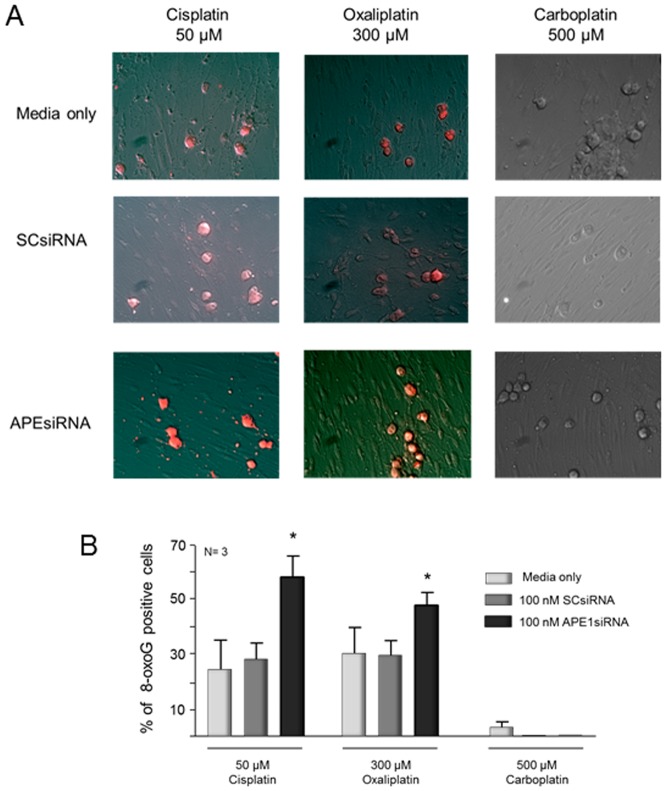
Platinum-induced oxidative DNA damage measured by 8-oxoG DNA adduct immunocytochemistry is increased by reducing APE1 expression in cisplatin and oxaliplatin treated sensory neuronal cultures. The top panels (A) show representative immunohistochemical staining for 8-oxoG DNA adducts in control (media), scrambled (SCsiRNA) or APE1 knockdown (APEsiRNA) from cultures after 24 hrs treatment with cisplatin (50 µM), oxaliplatin (300 µM) or carboplatin (500 µM). The bottom panels (B) represent the quantitation of the 8-oxoG positive staining cells as described in ”Methods.” The columns represent the mean ± SEM from cultures treated with media along (lightly shaded columns), SCsiRNA (medium shaded columns) or APE1siRNA (heavy shaded columns). An asterisk indicates a statistically significant increase in 8-oxoG in cells treated with APE1siRNA compared to those treated with SCsiRNA.

Further studies to demonstrate the relationship of platinum agent and oxidative DNA damage were performed using an antibody that detects 8-oxoguanine (8-oxoG) adducts in DNA [48]. Sensory neuronal cultures were treated with 50 µM cisplatin, 300 µM oxaliplatin, or 500 µM carboplatin. After 24 hrs, cisplatin and oxaliplatin caused a significant increase in 8-oxoG levels, 25 and 30% respectively, while carboplatin did not show any 8-oxoG production above background levels (Figure 4). When neuronal cultures were treated with APE1 siRNA, there was a 2-fold increase in the level of 8-oxoG with cisplatin treatment, while oxaliplatin showed a 1.5-fold increase compared to control cultures and cultures treated with SCsiRNA (Figure 4). Carboplatin did not induce any detectable levels of 8-oxoG regardless of APE1 status. These data are congruent with the results observed using the P-H2AX assay (Figure 3) and support our contention that oxidative DNA damage is induced by cisplatin and oxaliplatin, but not carboplatin.

### Oxaliplatin and carboplatin reduce capsaicin-evoked release of CGRP from sensory neurons

**Figure 5:**
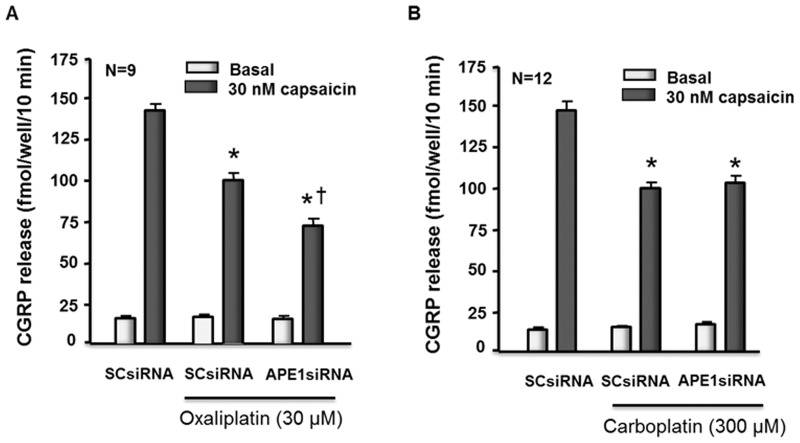
Reducing APE1 expression enhances the ability of oxaliplatin but not carboplatin to reduce capsaicin-evoked release of CGRP from sensory neurons in culture. Each column represents the mean ± SEM of CGRP release in fmol/well/min for untreated sensory neurons in culture (controls) or cultures treated with SCsiRNA or APE1siRNA as indicated. Cells were exposed to 30 µM oxaliplatin (A) or 300 µM carboplatin (B) for 24 hours prior to release experiments. For release, wells of cells from three independent harvests were exposed for 10 min to HEPES alone (basal; open columns), or HEPES in the presence of 30 nM capsaicin (solid columns) as indicated. An asterisk indicates a significant difference in capsaicin-stimulated release compared to untreated cells, whereas a cross indicates a significant difference in cultures treated with APE1siRNA versus those treated with SCsiRNA using Student's *t*-test. Cisplatin analyses can be found in our previous publication [24].

Previous studies by our group demonstrated that cisplatin decreases the release of the neuropeptide CGRP from sensory neurons, and that reducing APE1 expression augments this effect, while overexpressing APE1 attenuates the effect [7], [24]. The question remains whether oxaliplatin and carboplatin treatment also reduce transmitter release from sensory neurons and if altering APE1 expression produces the same effect on this endpoint of sensory neuronal function. When sensory neuron cultures that were transfected with SCsiRNA were exposed to 30 µM oxaliplatin or 300 µM carboplatin for 24 hours, there was a significant reduction in release of CGRP evoked by 30 nM capsaicin (Figure 5) with no change in basal release. In cultures not exposed to platins, capsaicin-evoked release was 141 ± 4 fmol/well/10 min and 143 ± 6 fmol/well/10 min, whereas after oxaliplatin or carboplatin release was 99 ± 5 and 97 ± 4 fmol/well/10 min, respectively. Reducing the expression of APE1 by transfecting cells with APE1siRNA augmented the oxaliplatin-induced decrease in CGRP release to 72 ± 4 fmol/well/10 min (Figure 5A). In contrast, reducing APE1 expression did not alter the ability of carboplatin to diminish CGRP release (Figure 5B; 100 ± 4 fmol/well/10 min).

### ROS generation in sensory neuronal cultures by platinum compounds

**Figure 6:**
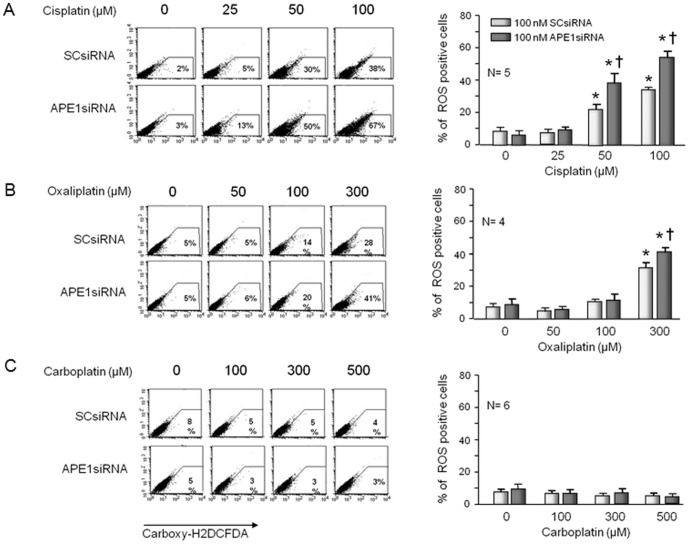
Production of reactive oxygen species (ROS) by cisplatin and oxaliplatin, but not carboplatin is increased by reducing the expression of APE1 in sensory neuronal cultures. Neuronal cultures were exposed to siRNAs on days 3-5 in culture then exposed to various concentrations of platins for 24 hours starting on day 11 in culture. ROS generation was measured by Carboxy-H2DCFDA and FACS analysis. The left panels show representative FACS for cells treated with various concentrations of cisplatin (A), oxaliplatin (B) or carboplatin (C) and scramble siRNA (SCsiRNA) or APE1siRNA as indicated. The panels on the right show the quantification of data from 4-6 independent harvests. Each column represents the mean ± SEM of the percent of ROS positive cells from cultures treated with SCsiRNA (lightly shaded) or with APE1siRNA (heavy shaded) and treated with various concentrations of cisplatin (A); oxaliplatin (B) or carboplatin (C) as indicated. An asterisk indicates significant difference in the number of ROS positive cells in the absence or presence of drug treatment, whereas a cross indicates significant difference in cultures treated with SCsiRNA versus APE1siRNA using Student's *t*-test.

The data presented above show that all three platinating agents have similar neurotoxic effects of sensory neurons as measured by cell death, DNA damage, and the diminished release of neurotransmitters. In the case of cisplatin and oxaliplatin, reducing APE1 expression augments the neurotoxicity, whereas with carboplatin, altering APE1 has little, if any, effect. Furthermore, we have previously demonstrated that the neurotoxicity produced by cisplatin is reversed by increasing APE1 repair activity [24]. These results suggest that cisplatin and oxaliplatin produce DNA damage that is susceptible to BER repair, whereas carboplatin does not. Previous studies have demonstrated production of ROS by cisplatin as well as its widely known DNA cross-linking effects [24], [49], [50]. Since ROS could result in DNA damage that is susceptible to BER, we asked whether cisplatin, oxaliplatin, or carboplatin could produce ROS in sensory neuronal cultures. Exposing sensory neuronal cultures transfected with SCsiRNA to 50 µM or 100 µM cisplatin for 24 hours significantly increased the number of ROS production as indicated by carboxy-H2DCFDA staining (Figure 6A), similar to what we have previously shown [24]. When transfecting APE1siRNA reduced APE1 expression, there was a significant increase in the amount of ROS production compared to those transfected with SCsiRNA (Figure 6A), again similar to previous studies [24]. Exposing cultures to 300 µM oxaliplatin for 24 hours, but not to lower concentrations, increased the percent of ROS production and this was significantly enhanced by reducing APE1 expression (Figure 6B). ROS production by carboplatin was not detected using carboxy-H2DCFDA even with doses up to 500 µM (Figure 6C). These data support the notion that sensory neuron dysfunction and therefore neuropathy secondary to cisplatin and oxaliplatin is due, at least in part, to ROS production and oxidative DNA damage that is acted upon by the BER pathway, particularly APE1. In contrast, carboplatin produces less dysfunction and DNA damage even at higher concentrations, which suggests that its toxicity is not influenced by the BER pathway.

**Figure 7:**
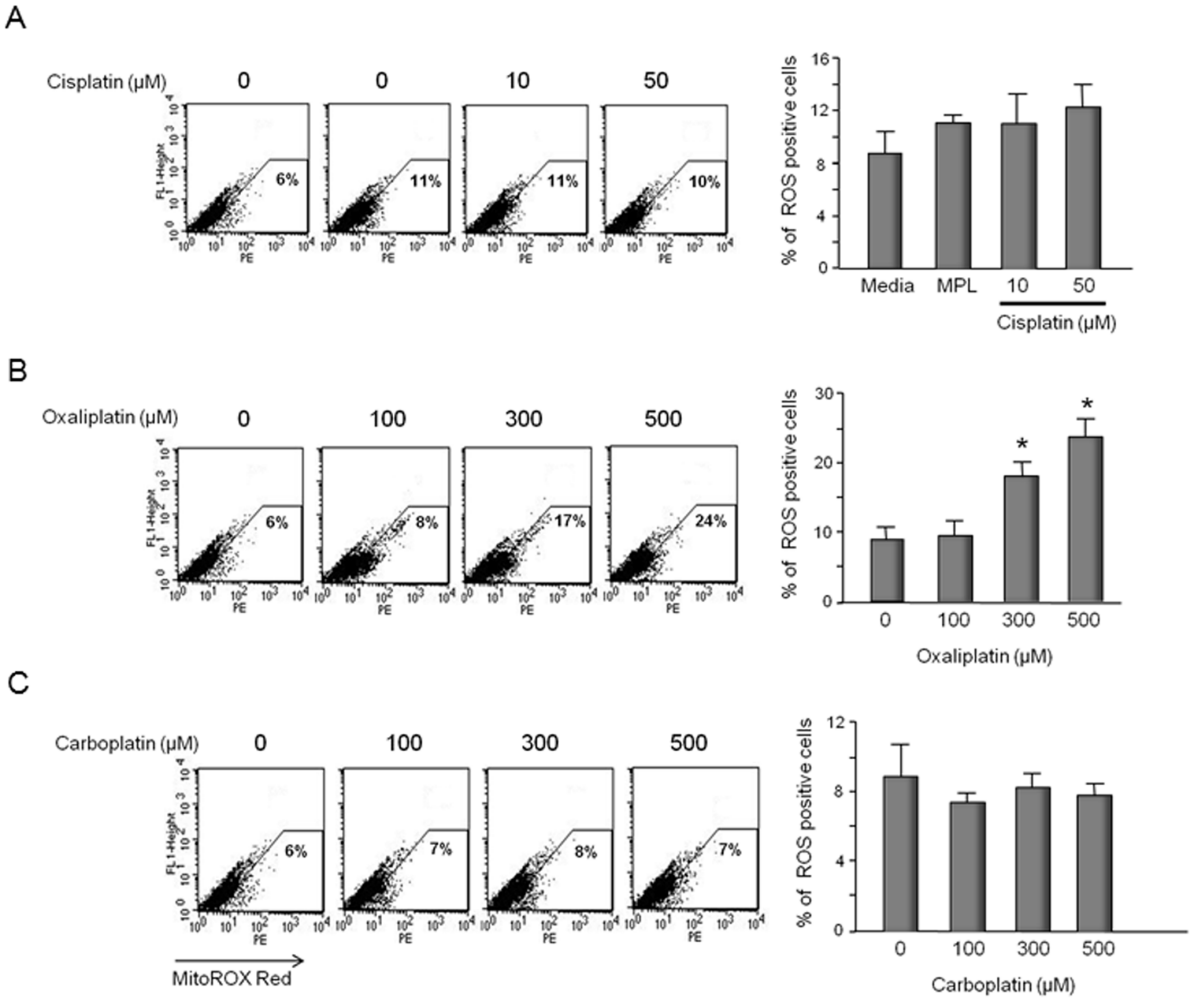
Oxaliplatin, but not cisplatin or carboplatin increase production of mitochondrial ROS in sensory neuronal cultures. Neuronal cultures were exposed to various concentration of cisplatin (A), oxaliplatin (B), or carboplatin (C) for 24 hours starting on day 11 in culture and mitochondrial ROS measured using MitoSOX red and FACS analysis. The left panels show representative FACS analysis for cells treated with various concentrations of cisplatin (A), oxaliplatin (B), or carboplatin (C) as indicated. The panels on the right show the quantification of data from three independent harvests. Each column represents the mean ± SEM of the percent of superoxide positive cells from cultures treated with various concentrations of cisplatin (A); oxaliplatin (B) or carboplatin (C) as indicated. An asterisk indicates significant difference in the number of superoxide positive cells compared to controls using Student's *t*-test.

To ascertain whether ROS production secondary to exposure to platins is more general in nature or specifically mitochondrial generated by superoxide, we performed a series of experiments using MitoSox Red which selectively targets mitochondria and is oxidized by superoxide anion, the predominant ROS in mitochondria, but not other ROS or reactive nitrogen species. Although total ROS positive cells were dramatically increased following 50 µM cisplatin (see Figure 6), there was no increase in the percent of cells producing mitochondrial ROS at this concentration (Figure 7A). At higher concentrations of cisplatin, however, mitochondrial ROS generation increased (see Figure S2). With oxaliplatin, 300 µM and 500 µM significantly increased the number of cells showing mitochondrial ROS production: 17% ROS positive cells at 300 µM and 24% at 500 µM (Figure 7B). In contrast, carboplatin at the concentrations tested did not increase the production of mitochondrial ROS in the neuronal cultures (Figure 7C). The ROS production in mitochondria after cisplatin treatment was not increased in cultures pretreated with APE1 siRNA to reduce expression of APE1 (see Figure S2).

In order to further to determine the production of ROS by the platinum agents, we investigated whether a general anti-oxidant scavenger, such as N-acetyl cysteine (NAC) would block the effects of cisplatin on sensory neuronal cultures. We determined that NAC blocks apoptosis in cultures following 50 µM cisplatin treatment (see Figure S3a), and it blocks total ROS production at both 50 and 100 µM cisplatin treatment levels (see Figure S3b). These data support our hypothesis that the platinating agents, and particularly cisplatin as an example, produce ROS as a function of their action as previously discovered in other model systems [24], [31], [51].

### Chronic administration of platinum agents reduces capsaicin-induced vasodilation in the rat hindpaw

**Figure 8:**
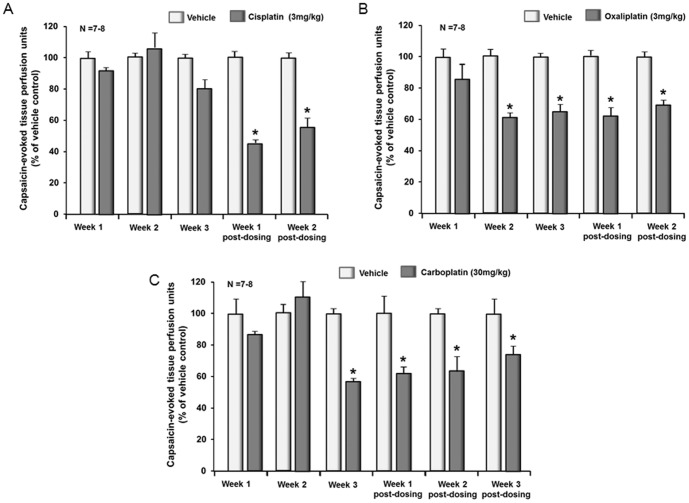
Systemic administration of cisplatin, oxaliplatin, or carboplatin decreases capsaicin-induced vasodilatation in the rat hindpaw. Each column represents the mean ± SEM of the capsaicin-evoked changes in blood flow over 15 minutes (capsaicin-stimulated blood flow minus basal blood flow) normalized to the vehicle treated controls. Animals are injected with vehicle (lightly shade columns) or platinum drugs (dark shaded columns) once a week for three weeks. Blood flow is measured each week 4 days after dosing with 3 mg/kg cisplatin (A), 3 mg/ml oxaliplatin (B) or 30 mg/kg carboplatin(C) and for 1–3 weeks after dosing is discontinued. An asterisk indicates statistical significance between the platinum-treated group and the corresponding vehicle-injected group using the statistical analysis software SPSS 11.0; post hoc analysis is LSD (Fisher's least significant difference) and Student-Newman-Keuls.

It has long been appreciated that activation of the peripheral endings of small diameter sensory nerves caused the release of neuropeptides that contribute to neurogenic inflammation [52]. Thus, measuring changes in peripheral blood flow after activation of sensory neurons provides a non-invasive and reproducible measure of the function of these neurons in the absence or presence of systemic administration of anticancer drugs [53]. Consequently, we used this technique to determine whether repeated administration of platinum drugs alters the function of sensory neurons *in situ*. When rats are administered 3 mg/kg cisplatin i.p once per week for three weeks there is a significant reduction in the vasodilation evoked by an intradermal injection of capsaicin which is not observed until one week after the last dose of drug and persists into week 2 post-dosing (Figure 8A; and see Figure S4 for raw data). Similar results are observed with a weekly administration of 3 mg/kg oxaliplatin, but the onset of the effect is first observed in the second week of drug dosing (Figure 8B). When rats received 10 mg/kg carboplatin weekly, there was no effect on capsaicin-evoked vasodilation (data not shown), but a diminished blood flow was observed after 30 mg/kg (Figure 8C). These data demonstrate that weekly administration of platinum drugs alters the function of small diameter sensory neurons *in situ* and confirm functional neurotoxicity of these anticancer drugs that could contribute to CIPN.

### Systemic administration of E3330 is neuroprotective against cisplatin-induced alterations in capsaicin-induced vasodilation

**Figure 9:**
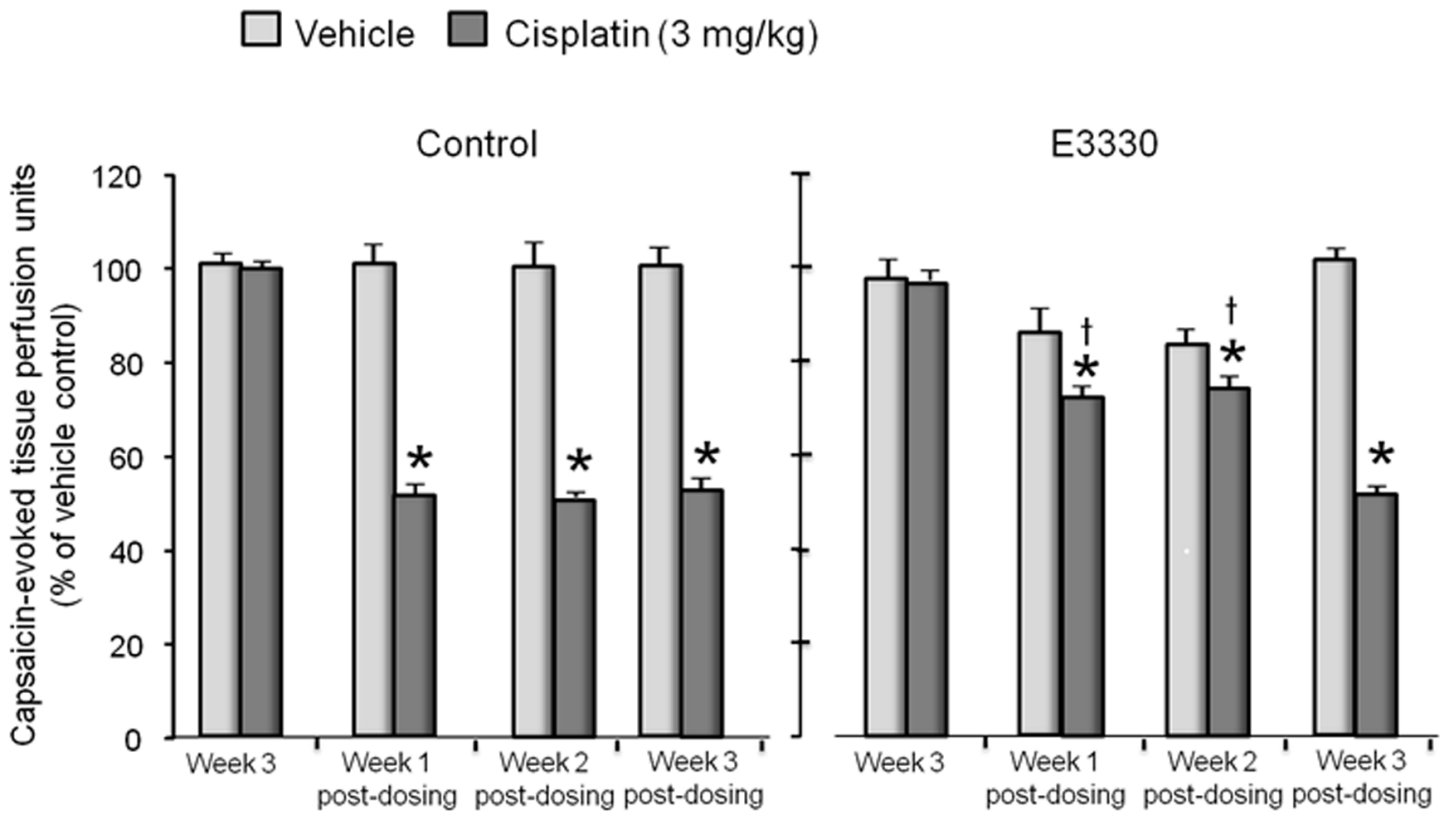
E3330 attenuates the cisplatin-induced decrease in capsaicin-induced vasodilatation in the rat hindpaw. Each column represents the mean ± SEM of the capsaicin-evoked changes in blood flow over 15 minutes (capsaicin-stimulated blood flow minus basal blood flow) normalized to the vehicle treated controls. Animals are injected with vehicle (lightly shade columns) or 3 mg/kg cisplatin (dark shaded columns) once a week for three weeks. The animals also were administered vehicle or E3330 (25 mg/kg) orally for five days each week for three weeks. Blood flow was measured at week three of dosing and each week for three weeks after cisplatin and E3330 treatments were discontinued. An asterisk indicates statistical significance between the animals treated with cisplatin and those treated with vehicle, whereas a cross indicates a significant difference in animals treated with cisplatin and E3330 versus those treated with just cisplatin.

Previous studies in our laboratory using isolated sensory neurons have shown that augmenting APE1 repair activity attenuates cisplatin-induced neurotoxicity in isolated sensory neurons [24]. Consequently, we examined whether augmenting APE1 activity *in situ* with the drug E3330 would also be neuroprotective. We chose to examine E3330 since we have previously shown in isolated sensory neurons that it prevents neurotoxicity caused by ionizing radiation [43]. Furthermore, when sensory neuronal cultures are exposed to E3330 for 24 hours, there is a concentration-dependent increase in APE1 repair activity (Figure S5). To determine whether E3330 was neuroprotective, we performed studies similar to those presented in Figure 8, but treated rats for three weeks with 5 daily doses of 25 mg/kg E3330 given orally and 3 mg/kg cisplatin given on day three of each week. When control rats were injected with cisplatin and vehicle controls, there was a significant decrease in the ability of capsaicin to induce vasodilation, which occurred one week post-dosing and was maintained for the additional two weeks of testing (Figure 9). In contrast, when rats were administered E3330 and cisplatin, there was no significant effect on capsaicin-induced vasodilation 1 week and 2 weeks post dosing (Figure 9). This protective effect was not observed at three weeks postdosing when E3330 was not present. However, there was a significant effect of cisplatin three weeks post dosing, which was similar to the effect observed in control rats. These data support the notion that enhancing the repair activity of APE1 is a viable approach to reversing cisplatin induced CIPN.

## Discussion

The data presented here further our earlier findings relating to the role of the BER pathway in altering the function of sensory neurons after exposure to cisplatin [24] by comparing the neurotoxicity caused by the three commonly used platinum agents: cisplatin, oxaliplatin, and carboplatin. We confirmed our previous work showing that cisplatin reduces cell viability, increases apoptosis, augments production of ROS, and increases 8-oxoG adducts at concentrations lower then we previously examined. We also demonstrated that oxaliplatin and carboplatin significantly reduced cell viability, but at concentrations substantially higher than cisplatin. With cisplatin and oxaliplatin, the reduced cell viability and increased apoptosis were further enhanced by APE1 knockdown. However, while carboplatin reduced cell viability, the effect was not enhanced by APE1 knockdown. As we previously observed with cisplatin for P-H2AX [24], oxaliplatin increased the amount of DNA damage as indicated by an increase in P-H2AX and 8-oxoG DNA adducts, and this effect was increased by reducing APE1 expression. Carboplatin exposure resulted in a small increase in P-H2AX compared to oxaliplatin with essentially no production of 8-oxoG DNA adducts. With a functional assay, exposure to both oxaliplatin and carboplatin decreased CGRP release evoked by capsaicin without altering resting release or total content of CGRP. The effect of these drugs on transmitter release is analogous to our previous results using cisplatin [24]. Of interest, although altering APE1 expression effects the inhibition of release by oxaliplatin (see Figure 4) and cisplatin (see [24]), reducing the expression of APE1 does not affect the actions of carboplatin.

It is well established that exposure of sensory neurons to various platinating agents results in the formation of adducts [19], and this formation correlates with platinum-induced neurotoxicity including apoptosis [20], [22], [27]. Since adducts are repaired largely by NER, this pathway is thought to be of major importance [54]. Our results, however, strongly support the notion that compromising the BER pathway worsens the neurotoxicity secondary to cisplatin and oxaliplatin, whereas with carboplatin, BER alteration does not seem to influence toxicity. It is interesting to speculate that one mechanism that could account for the augmented toxicity by cisplatin and oxaliplatin secondary to decreasing APE1 (i.e. reducing BER) is the observation that a 24-hour exposure to either cisplatin or oxaliplatin resulted in a significant production of ROS in the neuronal cultures, as well as 8-oxoG DNA adducts. This also would explain why reducing APE1 expression does not affect the actions of carboplatin, since this agent does not increase production of ROS nor DNA damage (8-oxoG). Moreover, the removal of APE1 from sensory neuronal cells resulted in an increase in ROS production with cisplatin and oxaliplatin, but had no effect with carboplatin. In the case of cisplatin, our results confirm our previous studies and those by others showing that this drug increases production of ROS [24], [31], [51]. Cisplatin-induced ROS production does not appear secondary to mitochondrial damage since at the concentrations which produce total ROS there is no increase in ROS in mitochondria. In contrast, oxaliplatin increases ROS in mitochondria at concentrations that increase total ROS in the neuronal cultures. Since oxidative DNA damage is repaired by the BER pathway [55] and because decreasing APE1 expression augments cisplatin and oxaliplatin toxicity, it seems likely that the oxidative DNA damage caused by these agents in the neuronal cultures is the determining factor for the observed effects. Interestingly, both cisplatin and carboplatin produce a similar Pt-1,2 (CpG) intrastrand DNA adduct which comprises greater than 90% of the crosslinks in the DNA [46], [56], yet there are clear differences between cisplatin and carboplatin in our studies, attributed to the amount of ROS produced (cisplatin and oxaliplatin) or not produced (carboplatin). These data are supported by our finding of 8-oxoG DNA adducts being produced by cisplatin and oxaliplatin, but not carboplatin and an augmentation of the 8-oxoG levels for the former two agents, but not the latter when APE1 levels are reduced.

We chose to examine the effects of the platinating agents on sensory neurons grown in culture, since the neuropathy that is observed after these drugs are given to patients is largely sensory and characterized by either a gain of function as observed with paraesthesia, allodynia and/or hyperalgesia, or a loss of function characterized by numbness, loss of reflexes, loss of proprioception, and/or cold intolerance in the extremities. It also is well established that systemic administration of either cisplatin or oxaliplatin result in platinum adduct formation in the cell bodies of sensory neurons [19], [20], [22]. Furthermore, previous work demonstrates that compromising either the BER or NER pathways in rodents worsens cisplatin-induced neurotoxicity [24], [27]. One interesting observation of examining the effects on sensory neurons in culture is that the level of toxicity correlates with the incidence of CIPN in patients. We observe a greater degree of toxicity at lower concentrations with cisplatin and oxaliplatin compared to carboplatin. This occurs with cell killing, DNA damage, and with production of ROS. Approximately 30–60% of patients administered cisplatin or oxaliplatin chronically develop significant CIPN [4], [6]–[8], whereas the number of patients developing CIPN during carboplatin therapy is much less (∼5% [16], [17]). One limitation of using sensory neuronal cultures, however is that we do not distinguish between the various subtypes of sensory neurons. For example, although we show apoptosis of cells in culture, we do not define whether they are large diameter or small diameter sensory neurons. Thus, we cannot correlate changes we see in the neuronal survival or production of ROS with functional changes seen *in vivo*. We do show that all three agents reduce the capsaicin-evoked release of CGRP, and this supports the notion that toxicity is occurring in small diameter peptidergic neurons and *in situ* these neurons comprise one group of nociceptors.

To begin to translate our findings in isolated sensory neurons to chronic dosing in animals, we employed a rat animal model to measure the function of peptidergic sensory neurons *in situ* [52], [53]. In this model, after long-term administration we measured the ability of the platinum drugs to alter capsaicin-evoked vasodilatation which is regulated by release of CGRP from the peripheral endings of small diameter sensory neurons [53], [57]. While each chemotherapeutic agent had a slightly different pattern of effect, all three caused a significant reduction in capsaicin-induced vasodilatation that was delayed in onset. In the case of cisplatin, the effect was not observed until after stopping the three weeks of dosing, whereas with oxaliplatin, the onset was after the second dose of the drug. The inhibitory effect of carboplatin was observed during week three of dosing. In all cases, the effect of the drugs was maintained for 2–3 weeks after dosing was discontinued. This pattern of drug toxicity is analogous to the time course for the development of CIPN in patients in that the onset of neuropathy is delayed and is often maintained after therapy is discontinued [4], [7]–[9]. It also is important to note that the dose of carboplatin necessary to produce the inhibition of capsaicin-induced vasodilatation is 10 times higher than that of cisplatin and oxaliplatin. Although not shown, a dose of 10 mg/kg given weekly for three weeks did not alter the capsaicin-induced vasodilatation, which is consistent with the fact that carboplatin does not produce CIPN in most patients treated with the drug [16], [17].

When rats were treated systemically with the redox inhibitor of APE1, E3330, the inhibitory effects of cisplatin on capsaicin-induced vasodilatation were significantly attenuated for the first two weeks after dosing was stopped, suggesting that this compound protects the sensory neurons. Given our previous studies suggesting that the neuroprotective effects of APE1 on sensory neurons are secondary to enhanced repair, not to its redox action [24], [43], the effect of E3330 seems at first difficult to explain. Although E3330 has been shown to attack the primary redox cys in APE1, cys65, affecting its ability to be reduced and preventing APE1 redox activity, it also disrupts the interaction of cys65 and the other two cys that interact with cys65, namely cys93 and cys99, mainly through an unfolding of the APE1 protein over time and reducing the ability of disulfide bonds to be formed. [58]–[61]. This unfolding primarily alters the amino end of APE1, where the redox function and APE1 protein-protein interaction regions are located and releases APE1 from its redox activities and potentially facilitates APE1 repair activity. *In vivo* this could translate to a protective mechanism facilitating BER to repair damage induced by cisplatin. Indeed, when isolated sensory neurons are exposed to E3330, there is a concentration-dependent increase in APE1 endonuclease activity (Figure S4). Furthermore, a similar result was observed *in vitro* where E3330 was shown to protect sensory neurons from damage induced by ionizing radiation [43]. While more detailed studies on the mechanism by which E3330 is neuroprotective are necessary, this data is encouraging as it demonstrates that targeting APE1 by enhancing its DNA repair capability is a viable approach to reversing cisplatin induced CIPN.

To date, the mechanisms by which anticancer drugs produce peripheral neuropathy remain unknown, although several mechanisms have been proposed. These include changes in the target regions of sensory nerve endings in the periphery, alterations in mitochondrial function in neurons, and/or changes in the immune/neuronal interaction. In animal models, several observations have been made correlating chemotherapy-induced neuropathic pain with changes in mitochondrial function in neurons [62], [63], with changes in the immune system which can alter neuronal function [64], [65], and with alterations in the morphology of neurons [63], [65]. No causal relationship has been established between these changes and the initiation and maintenance of CIPN. Our studies suggest another mechanism for CIPN: DNA damage which could alter the function of sensory neurons in ways that manifest as the various symptoms observed in CIPN [66]. Clearly, exposing sensory neurons to anticancer drugs produces DNA damage (see Figure 3 and [20], [22], [41]), and previous models have suggested that damage resulting from single strand breaks, such as what would occur if ROS adducts were not properly or completely repaired, would lead, in non-proliferating cells, to block transcription and lead to deficient neuronal proteins and dysfunction or cell death [67].

If one assumes that CIPN occurs secondary to DNA damage, then DNA repair in neurons could be a critical way of preventing the CIPN that occurs in patients. Our results with E3330 support this notion, as do our previous studies using overexpression of APE1 to protect neurons from cisplatin-induced toxicity. Other reports also have implicated the BER pathway in modulating and repairing cisplatin DNA damage by synthesizing past interstrand adducts, while further studies have demonstrated that the interstrand cross-links can be substrates of the BER pathway [68]. Moreover, we have previously demonstrated a relationship between APE1, p53, and GADD45a in response to cisplatin damage correlating with ROS in sensory neuronal cultures. The p53-GADD45a pathway is also intimately linked to the repair of DNA damage related to cross-linking agents such as platinum that are normally repaired by the NER pathway [24], [66]. Therefore, it appears there is a clear and intimate role of BER and particularly APE1 in protecting sensory neurons from the toxic effects of cisplatin and oxaliplatin relating to CIPN, and most likely coordination between the BER and NER pathways. This latter link is also currently under investigation in our laboratory.

In conclusion, we demonstrate that the loss of APE1 function increases the sensitivity of sensory neuronal cultures to cisplatin and oxaliplatin for both cell survival and function, but not carboplatin, and correlates with the level of DNA damage induced by these agents as well as ROS levels produced. We also demonstrate differences in the platinum agents on peripheral neuropathy as defined by peripheral blood flow *in vivo* using a rat model, and we demonstrate the *in vivo* use of a small molecule targeting APE1 which shows protective activity against cisplatin-induced neuropathy.

## Materials and Methods

### Ethics Statement

Animals used in these studies and the experimental protocols were approved by the Indiana University School of Medicine Institutional Animal Care and Use Committee (IACUC), # 10119.

### Materials

Unless otherwise specified, tissue culture supplies were obtained from Invitrogen (Carlsbad, CA). Poly-D-lysine, laminin, and routine chemicals were purchased from Sigma-Aldrich (St. Louis, MO). Nerve growth factor was purchased from Harlan Bioproducts for Science (Indianapolis, IN) and Normocin from Invivogen (San Diego, CA). Neuroporter were purchased from Genlantis (San Diego, CA). Mouse monoclonal antihuman APE1 antibodies were raised in our laboratory and available from Novus Biologicals (Littleton, CO), whereas the mouse monoclonal anti-phospho-H2AX antibodies were from EMD Millipore (Billerica, MA) and β-Actin monoclonal antibody from Thermo Fisher Scientific (Fremont, CA). Chemiluminescence secondary antibodies were from Roche Diagnostics Corp. (Indianapolis, IN). Alexa Fluor 488 Annexin-V Vybrant Apoptosis Assay Kits were from Molecular Probes (Eugene, OR, USA) and chemiluminescence (Roche Diagnostics Corp., Indianapolis, IN).

E3330 was synthesized per previous publications [41], [69], dissolved in N,N-dimethylformamide (Sigma-Aldrich) and stored as a 40mM at −80°C. For oral administration, E3330 was diluted to the appropriate amount in vehicle composed of Cremophor E::EtoH (1∶1), 4% total volume with saline [70]. Cisplatin was purchased from Sigma-Aldrich Inc. (St. Louis, MO), dissolved in 1-methyl-2-pyrrolidone (Sigma-Aldrich) and stored as a 50mM at −20°C for a month. Oxaliplatin was purchased from LKT Laboratories, Inc., dissolved in PBS, and stored as a 5mM stock at −80°C. Carboplatin was purchased from Sigma-Aldrich Inc., dissolved in culture medium, and stored as a 20mM stock at −80°C. Before drug treatment, the stocks were diluted in F-12 growth medium and added to cultures and incubated for 8–72 hours as indicated. The Animal Care and Use Committee at Indiana University School of Medicine, Indianapolis, IN approved all procedures used in these studies.

### Cell culture

Dorsal root ganglia (DRG) were dissected from all spinal levels of adult male (150–175 g) Sprague-Dawley rats (Harlan, Indianapolis, IN) and the cells were dissociated as previously described [71]. Briefly, the rats were euthanized by CO_2_ asphyxiation. DRGs were transferred into collagenase solution (1 mg/ml) and incubated for 1hr at 37°C. The digested DRGs were then rinsed with growth medium, centrifuged and dissociated by mechanical agitation. Approximately 30,000 cells or 60,000 cells were plated into each well of 12-well or 6-well culture plates, respectively. All culture dishes were precoated with poly-D-lysine and laminin. Cells were maintained in F-12 media supplemented with 10% horse serum, 2 mM glutamine, 100 µg/ml Normocin™, 50 µg/ml penicillin, 50 µg/ml streptomycin, 50 µM 5-fluoro-2′-deoxyuridine (Invitrogen), 150 µM uridine, and 30 ng/ml of NGF in 3% CO_2_ at 37°C. Growth medium was changed every other day.

### Reducing Ape1 expression using small interfering RNA

Small interfering RNAs to APE1 (APE1siRNA) and scrambled siRNA (SCsiRNA) controls were used to decrease APE1 protein expression in sensory neuronal cell cultures and as controls, respectively, as described previously [24], [71] On day 3 in culture, the growth media was replace with 0.5 ml of Opti-MEM 1 media containing 100 nM of APE1siRNA (5′-GUCUGGUAAGACUGGAGUACC-3′) or SCsiRNA (5′-CCAUGAGGUCAGCAUGGUCUG-3′;[71]) and 10 µl of the transfecting reagent, Neuroporter. On the next day, 0.5 ml of the growth media without antibiotics was added to each well, and after an additional 24 hours the media containing siRNA was replaced with normal growth media.

### APE1 activity assay

The APE1 activity assay was performed as previously described [72] and used for data presented in Figure S1 [73], [74]. Briefly, 6.25ng of the protein extracts from neuronal cultures were added to the reaction mix containing assay buffer (50mM HEPES, pH 7.5, 50mM KCl, 1mM MgCl_2_, and 2mM DTT) and 25nM HEX-labeled and a 26-base pair oligonucleotide substrate containing a THF moiety at position 13 in a total reaction volume of 20 µl. The reaction mixture then was incubated at 37°C for 30 min, and the reaction was stopped by the addition of 10 µl of formamide. Then 20 µl of the APE1 assay products were separated on a 20% denaturing (7M urea) polyacrylamide gel in 1×Tris-borate EDTA at 300V for 35 min to reveal two bands: the longer full-length labeled strand and the shorter cleaved fragment with the HEX label. To normalize the amount of immunoreactivity, 5 µg of each sensory neuronal cell protein extract sample was used to determine actin levels and density of the oligonucleotide bands normalized to the density of the actin bands.

The effects of E3330 on APE1 DNA repair activity were measured as previously described using the high-throughput screen (HTS) for APE1 inhibitors published by us and others and shown in Figure S4 [72], [75]-[77]. Briefly, the APE1 repair activity assay was performed in a plate assay using two annealed oligonucleotides (5′-6-FAM-GCCCCC*GGGGACGTACGATATCCCGCTCC-3′ and 3′-Q-CGGGGGCCCCCTGCATGCTATAGGGCGAGG-5′) custom synthesized by Eurogentec Ltd. (Belgium). The oligonucleotides contained a quencher on one strand and a fluorescent 6-FAM label with an AP site mimic, tetrahydrofuran (*), on the complimentary strand. The AP site mimic is a direct target of APE1's repair function. Cleavage of the oligo at this site results in release of the 6-FAM portion of the oligo from the complimentary strand with the quencher. The amount of fluorescence due to this cleavage is directly proportional to APE1's repair activity. Sensory neuronal cell cultures were treated with each dose of the compound in four separate experiments. The cell extracts from each experiment were run in triplicate for each 96-well plate assay in a 200uL volume for a total of 12 replicates of each treatment. A master mix was made that would provide a final amount of 50nM annealed oligo, 50mM TRIS, 1mM MgCl_2_ and 50mM NaCl, pH 7.5 in 150uL in each well. Two ug of each cell extract was added in a volume of 50uL to the appropriate wells, then immediately assayed. The fluorescence was read at five, one-minute intervals using a Tecan Ultra plate reader (Chemical Genomics Core, Indiana University School of Medicine). The rate of the reaction was used to determine the change in APE1 repair activity as compared to the vehicle control.

### Cell viability and apoptosis assays

Trypan blue exclusion analysis was performed as previously described [43]. Briefly, cells were detached from the plate using a 0.05% trypsin-EDTA solution and media was added. Equal volumes of the cell suspension and 0.4% (w/v) trypan blue in PBS were mixed, and the cells were scored under a phase contrast microscope using a hemacytometer. Percent survival was calculated as the percent of live cells divided by the total cell number (including dead and live cells).

Flow cytometric detection of apoptosis was performed using the Alexa Fluor 488 Annexin-V Vybrant Apoptosis Assay Kit in combination with propidium iodide (PI) according to manufacturer's instructions. Cells were harvested using Trypsin-EDTA. After washing the cells twice with PBS, they were resuspended in 100 µl binding buffer and stained with Annexin V-FITC/propidium iodide (PI). The cell suspension was incubated for 15 min in the dark at 4°C. The percentage of cells undergoing apoptosis was determined using flow cytometry. Apoptotic cells were defined as those positive for Annexin V.

### ROS detection assays

Sensory neuronal cultures were treated with cisplatin, oxaliplatin, and carboplatin at various concentrations, washed with PBS, then incubated with incubated with 10-µmol/L carboxy-H_2_DCFDA (Invitrogen) in fresh PBS for 60 min to assay total ROS. Excessive probe was washed off with PBS. The cells were harvested with trypsin, washed with PBS twice, and then suspended in 500 µl PBS. The fluorescence of the labeled cells was measured by using a Coulter EPICS XL flow cytometer (Coulter) with a fluorescence excitation of 485 nm and emission at 538 nm.

To assay mitochondrial superoxide, a 1 µmol/l MitoSOX reagent working solution in HBSS/Ca/Mg buffer was made fresh. One ml of this reagent was applied added to each well of cells and the cultures incubated for 10 min at 37°C. Cultures were washed with HBSS/Ca/Mg buffer, then the cells were trypsinized and harvested. Cells were washed twice with HBSS/Ca/Mg buffer then suspended in 500 µl HBSS/Ca/Mg buffer. The fluorescence of the labeled cells was measured by using a Coulter EPICS XL flow cytometer (Coulter) with a fluorescence excitation of 510 nm and emission at 580 nm. An average of 10,000 cells from each sample was counted, and each experiment was done at least in triplicate.

### 8-oxoguanine DNA damage assay

Measurement of 8-oxyguanosine immunoreactivity was performed using a modification of the method of Kinoshita and co-workers [48]. After 10 days in culture, neurons treated with siRNAs or vehicle on days 3-5 were exposed to platinum compounds for 24 hours then washed with PBS and fixed for 5 min at 4°C with an ethanol: methanol (1∶1; v/v) solution. Following three washes with PBS, cells on plates were exposed to RNase A (DNase and Protease-free) solution (200ug/ml; Thermo Scientific, EN0531) for 60 minutes to deplete RNAs. After a brief wash with PBS, fixed cells were incubated overnight at 4°C in primary antibody solution containing mouse anti-8-oxoguanine monoclonal IgM (1∶1000; Abcam), 5% normal donkey serum, 0.1% triton X 100 and 0.02% sodium azide in PBS, followed by a brief PBS wash and incubation with secondary antibody solution containing Alexa Fluor-conjugated goat anti-mouse IgM (1∶1000; Invitrogen) for 2 hrs. Cells then were washed with PBS for 30 minutes and viewed under a Nikon Eclipse Ti-S fluorescence microscope. Images were acquired using a Qimaging QICAM color camera and Qcapture Pro 6.0 Image Processor software (Qimaging, British Columbia, Canada). Bright field and fluorescent images from the same spot were taken, and three randomly selected spots were counted for each experimental condition by personnel blinded to the treatment. Digital images were visualized using Adobe Photoshop CS5 (Adobe System Inc., San Jose, CA).

### Immunoblotting

Cells were harvested, lysed in RIPA buffer (Santa Cruz Biotechnology; Santa Cruz, CA, USA). Protein was quantified using Lowey assay, and electrophoresed in a 12% SDS-polyacrylamide gel. After electrophoresis, the gel was transferred to a PVDF membrane, and blocked with Tris-buffered saline containing 0.1% Tween-20 (TBST) and 5% nonfat dry milk for 1 h at room temperature while gently agitating. Mouse monoclonal antihuman Ape1 antibodies (1∶1000), mouse monoclonal anti-phospho H2AX antibodies (1∶1000), or β-Actin monoclonal antibody (1∶1000) were added to the blocking solution and incubated for 2 h at room temperature while gently agitating. Antibody binding was detected following appropriate secondary antibody methods using chemiluminescence. The density of the bands was measured using QualityOne software from Bio-Rad (Hercules, CA) and data expressed as density normalized to actin.

### Measurement of CGRP Release

After neuronal cultures were treated with the appropriate drugs, the cultures were washed once with HEPES buffer consisting of (in mM) 25 HEPES, 135 NaCl, 3.5 KCl, 2.5 CaCl2, 1 MgCl2, 3.3 D-glucose, and 0.1% bovine serum albumin, pH 7.4 and maintained at 37°C. They were then incubated for successive 10 min intervals with 0.4 ml of HEPES buffer alone (basal release), with buffer containing 30 nM capsaicin, then with buffer alone (to assess return to basal release). After each incubation, the buffer was removed and the amount of immunoreactive CGRP in each sample was measured using radioimmunoassay as previously described (Chen et al., 1996). After the release experiment, the cells in each well were in 0.4 ml of 0.1 M HCl 10 min and an aliquot taken to measure total CGRP content in the cultures using radioimmunoassay. Total content (fmol/well) was calculated by adding the total amount released in all incubations to the amount measured in the cells. The release data is calculated as fmol released/well/10 min.

### Measurement of alterations in blood flow

Blood flow in the rat hindpaw was measured as previously described (Gracias et al., 2011). Rats were anesthetized with 100 mg/kg sodium thiopental and the hair on the dorsal hindpaw shaved and placed on a heated (37°C) platform to maintain body temperature. Blood flow was measured using a BLF21D laser Doppler flowmeter from Transonic systems Inc. (Ithaca, NY), and a type N 11 G needle-style probe gently placed in contact with the hindpaw using a micromanipulator. This system measures activity of red blood cell flux in ∼ 2 mm^3^ area beneath the probe [78]. Voltage output corresponding to tissue perfusion units (TPUs) were recorded on-line using Biopac data acquisition system (Goleta, CA). Vasodilatation was induced by intradermal injection of 2 µl of a 0.01% 1-methyl-2-pyrrolidinone solution (Aldrich Chemical Co., Milwaukee, WI) containing 10 µM capsaicin (Sigma Chemical Company, St. Louis, MO). Injections were made 1 mm away for the probe site. Baseline blood flow was measured for 12 min prior to capsaicin injection and evoked blood flow measured for 27 min after the capsaicin injection. The data for blood flow experiments were recorded per 3-minutes as tissue perfusion units (TPU) for each animal. Evoked response was calculated by subtracting the baseline response/15 minutes from the stimulated response/15 minutes.

### Statistical analysis

Data is expressed as the mean ± SEM from at least three repeats of each experiment. Differences in cell survival using trypan blue exclusion, P-H2AX, and CGRP release were determined using two-way analysis of variance (ANOVA) and Tukey's post hoc test. Differences in apoptosis or in the number of cells with ROS staining were determined using Student t-tests. Differences in blood flow were determined using one-way ANOVA and Bonferroni's *post hoc* test. In all cases, significance was set at *p* < 0.05) comparing treated versus controls. For the animal studies, statistical significance between the platinum-treated group and the corresponding vehicle-injected group was performed using the statistical analysis software SPSS 11.0; post hoc analysis is LSD (Fisher's least significant difference) and Student-Newman-Keuls.

## Supporting Information

Figure S1 **Treating sensory neuronal cultures with APE1siRNA significantly reduces APE1 expression and endonuclease activity.** A: The top panel shows a representative Western blot of APE1 and actin from neuronal cultures exposed to 100 nM scrambled siRNA (SCsiRNA) or 100 nM APE1siRNA on days 3–5 in culture and measured after 12 days in culture. The panel at the bottom shows the mean ± SEM of the density of the APE1 bands normalized to the amount of actin from three independent harvests of cells treated with SCsiRNA or APE1si RNA as indicated. B: The top portion of the figure shows a representative Western blot demonstrating endonuclease activity of Ape1 as indicated by the relative density of the 26 mer and 14-mer bands and actin (as a loading control) for extracts of cultures exposed to SCsiRNA or APE1siRNA as indicated using one of two routine AP endonuclease assays established in our laboratory [72]–[74]. The panel at the bottom shows the mean ± SEM of the percent cleavage of the 26 mer band normalized to the amount of actin from three independent harvests of cells treated with SCsiRNA or APE1si RNA as indicated. An asterisk indicates a statistically significant difference between SCsiRNA treated and APE1siRNA treated cells using Student's *t*-test.
(TIF)

Figure S2 **Reducing APE1 expression does not augment the ability of cisplatin to produce mitochondrial ROS in sensory neuronal cultures.** Neuronal cultures were exposed to siRNAs on days 3–5 in culture then exposed to various concentrations of cisplatin for 24 hours starting on day 11 in culture. Mitochondrial ROS was measured using MitoSox red and FACS analysis. The panels show representative FACS analysis for cells treated with various concentrations of cisplatin as indicated. The number in each box is the percentage of fluorescence positive cells.
(TIF)

Figure S3 **Effect of a general anti-oxidant n-acetyl cysteine (NAC) on ROS production in sensory neuronal cultures following cisplatin treatment.** ROS generation was measured by Carboxy-H2DCFDA and FACS analysis as in Figure 6. Neuronal cultures were exposed to various concentrations of NAC and 50 uM cisplatin for 24 hours starting on day 11 in culture. (A) Cell apoptosis was detected by Annexin-V and PI staining and FACS analyses. Numbers in the upper right box indicate the number of Annexin/PI positive cells. The panels in (B) show the level of ROS production following cisplatin treatment for 24 hrs at 0, 50 or 100 µM and NAC at 0, 5 or 10 mM concentrations. The numbers in the box are the carboxy-H2DCFDA positive cells.
(TIF)

Figure S4 **Capsaicin-induced cutaneous vasodilatation is attenuated two weeks after dosing of systemic cisplatin is discontinued.** Each column is the mean ± SEM of the tissue perfusion units/3 minutes in six rats treated with 3 mg/kg cisplatin once a week for three weeks. The light-shaded columns represent the basal blood flow and the dark-shaded columns represent blood flow after injection of 10 µM capsaicin as indicated. The top panel shows blood flow three days after the first injection of cisplatin, the middle panel three days after the third injection, and the bottom panel blood flow two weeks after stopping drug administration.
(TIF)

Figure S5 **Treating sensory neuronal cultures with E3330 significantly increases APE1 endonuclease activity.** Each column is the mean ± SEM of the percent increase in APE1 endonuclease activity using the established AP endonuclease assay (see methods). Activity was measured for extracts of cultures exposed to vehicle control or various concentrations of E3330 for 24 hours as indicated. An asterisk indicates a statistically significant difference between cultures treated with vehicle and those treated with E3330 using Student's *t*-test.
(TIF)
