# Supplementary figures and images for: Role of the DNA Base Excision Repair Protein, APE1 in Cisplatin, Oxaliplatin, or Carboplatin Induced Sensory Neuropathy
Source: PLoS One. 2014 Sep 4;9(9):e106485. doi: 10.1371/journal.pone.0106485 (PMC4154694; doi:10.1371/journal.pone.0106485)

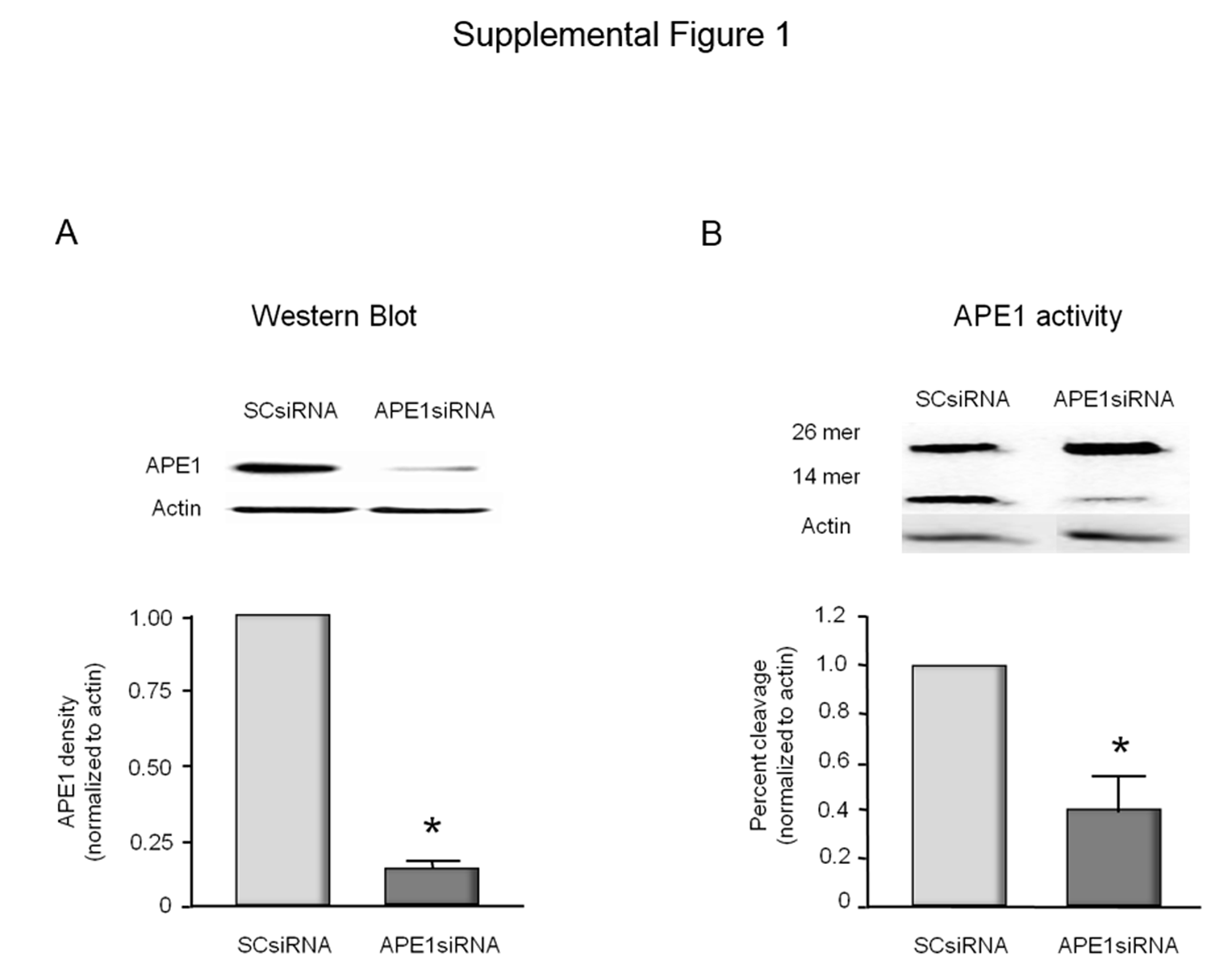

Supplement: Figure S1 — Treating sensory neuronal cultures with APE1siRNA significantly reduces APE1 expression and endonuclease activity. A: The top panel shows a representative Western blot of APE1 and actin from neuronal cultures exposed to 100 nM scrambled siRNA (SCsiRNA) or 100 nM APE1siRNA on days 3–5 in culture and measured after 12 days in culture. The panel at the bottom shows the mean ± SEM of the density of the APE1 bands normalized to the amount of actin from three independent harvests of cells treated with SCsiRNA or APE1si RNA as indicated. B: The top portion of the figure shows a representative Western blot demonstrating endonuclease activity of Ape1 as indicated by the relative density of the 26 mer and 14-mer bands and actin (as a loading control) for extracts of cultures exposed to SCsiRNA or APE1siRNA as indicated using one of two routine AP endonuclease assays established in our laboratory [72]–[74]. The panel at the bottom shows the mean ± SEM of the percent cleavage of the 26 mer band normalized to the amount of actin from three independent harvests of cells treated with SCsiRNA or APE1si RNA as indicated. An asterisk indicates a statistically significant difference between SCsiRNA treated and APE1siRNA treated cells using Student's t-test. (TIF) [file pone.0106485.s001.tif]

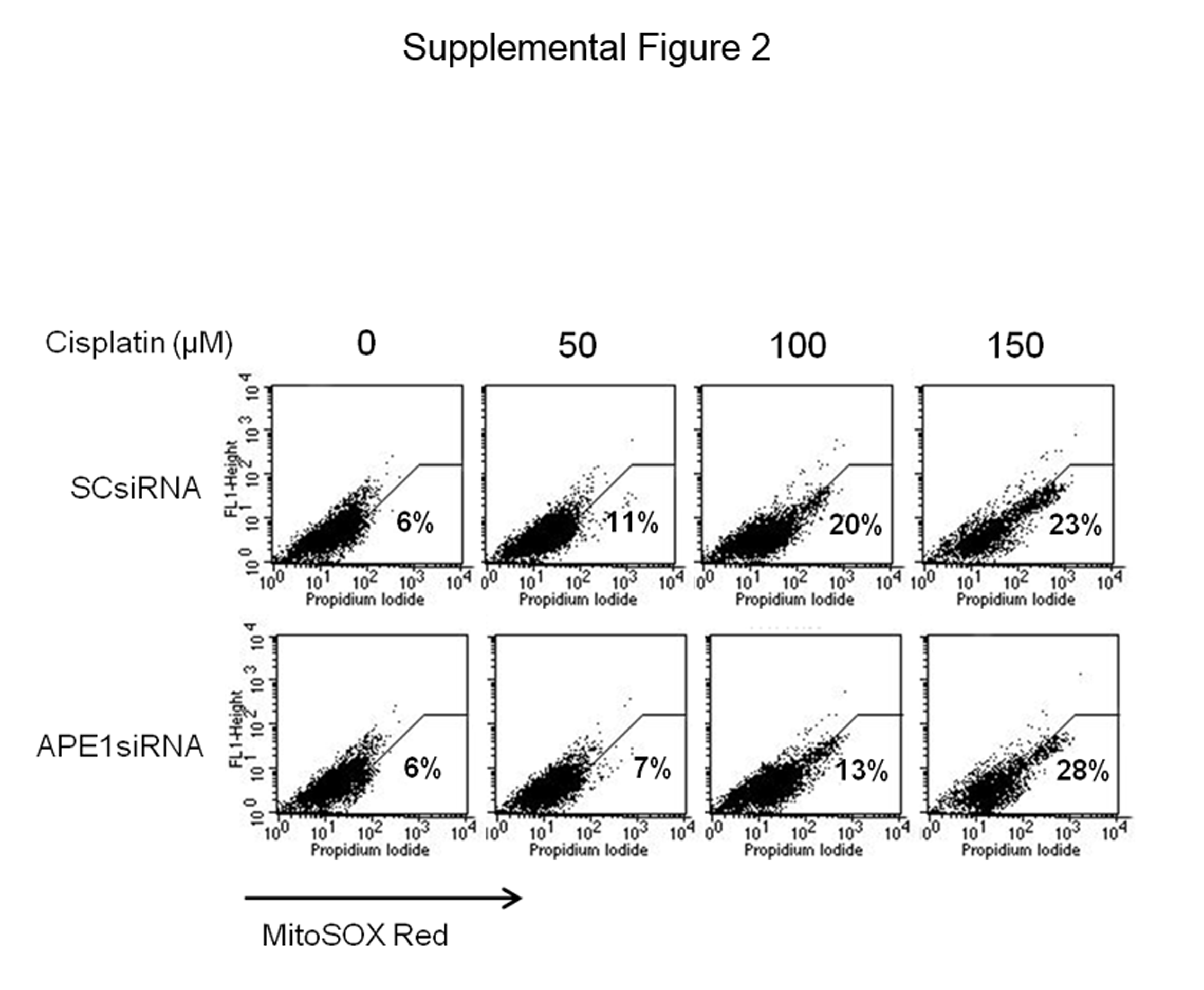

Supplement: Figure S2 — Reducing APE1 expression does not augment the ability of cisplatin to produce mitochondrial ROS in sensory neuronal cultures. Neuronal cultures were exposed to siRNAs on days 3–5 in culture then exposed to various concentrations of cisplatin for 24 hours starting on day 11 in culture. Mitochondrial ROS was measured using MitoSox red and FACS analysis. The panels show representative FACS analysis for cells treated with various concentrations of cisplatin as indicated. The number in each box is the percentage of fluorescence positive cells. (TIF) [file pone.0106485.s002.tif]

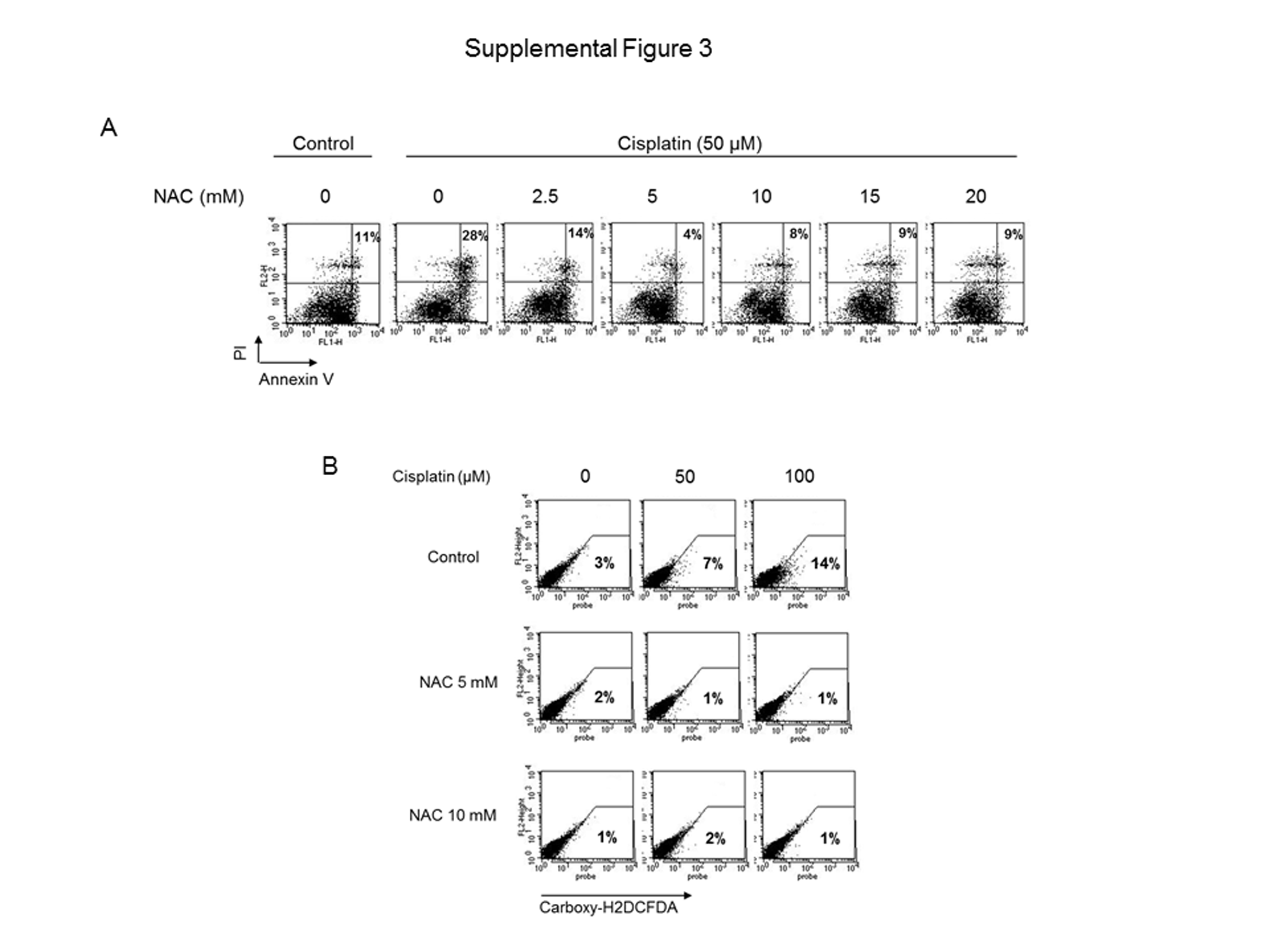

Supplement: Figure S3 — Effect of a general anti-oxidant n-acetyl cysteine (NAC) on ROS production in sensory neuronal cultures following cisplatin treatment. ROS generation was measured by Carboxy-H2DCFDA and FACS analysis as in Figure 6. Neuronal cultures were exposed to various concentrations of NAC and 50 uM cisplatin for 24 hours starting on day 11 in culture. (A) Cell apoptosis was detected by Annexin-V and PI staining and FACS analyses. Numbers in the upper right box indicate the number of Annexin/PI positive cells. The panels in (B) show the level of ROS production following cisplatin treatment for 24 hrs at 0, 50 or 100 µM and NAC at 0, 5 or 10 mM concentrations. The numbers in the box are the carboxy-H2DCFDA positive cells. (TIF) [file pone.0106485.s003.tif]

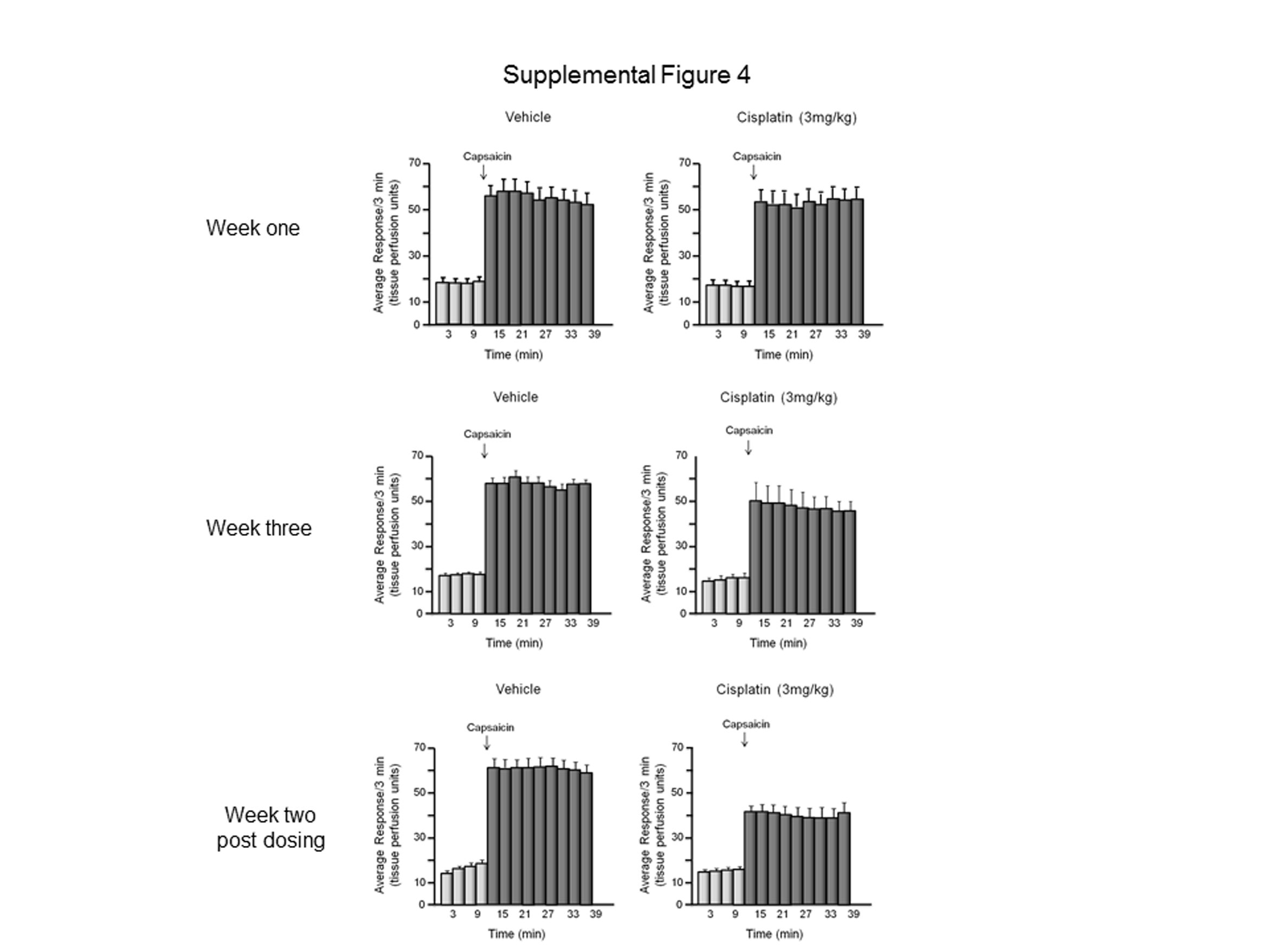

Supplement: Figure S4 — Capsaicin-induced cutaneous vasodilatation is attenuated two weeks after dosing of systemic cisplatin is discontinued. Each column is the mean ± SEM of the tissue perfusion units/3 minutes in six rats treated with 3 mg/kg cisplatin once a week for three weeks. The light-shaded columns represent the basal blood flow and the dark-shaded columns represent blood flow after injection of 10 µM capsaicin as indicated. The top panel shows blood flow three days after the first injection of cisplatin, the middle panel three days after the third injection, and the bottom panel blood flow two weeks after stopping drug administration. (TIF) [file pone.0106485.s004.tif]

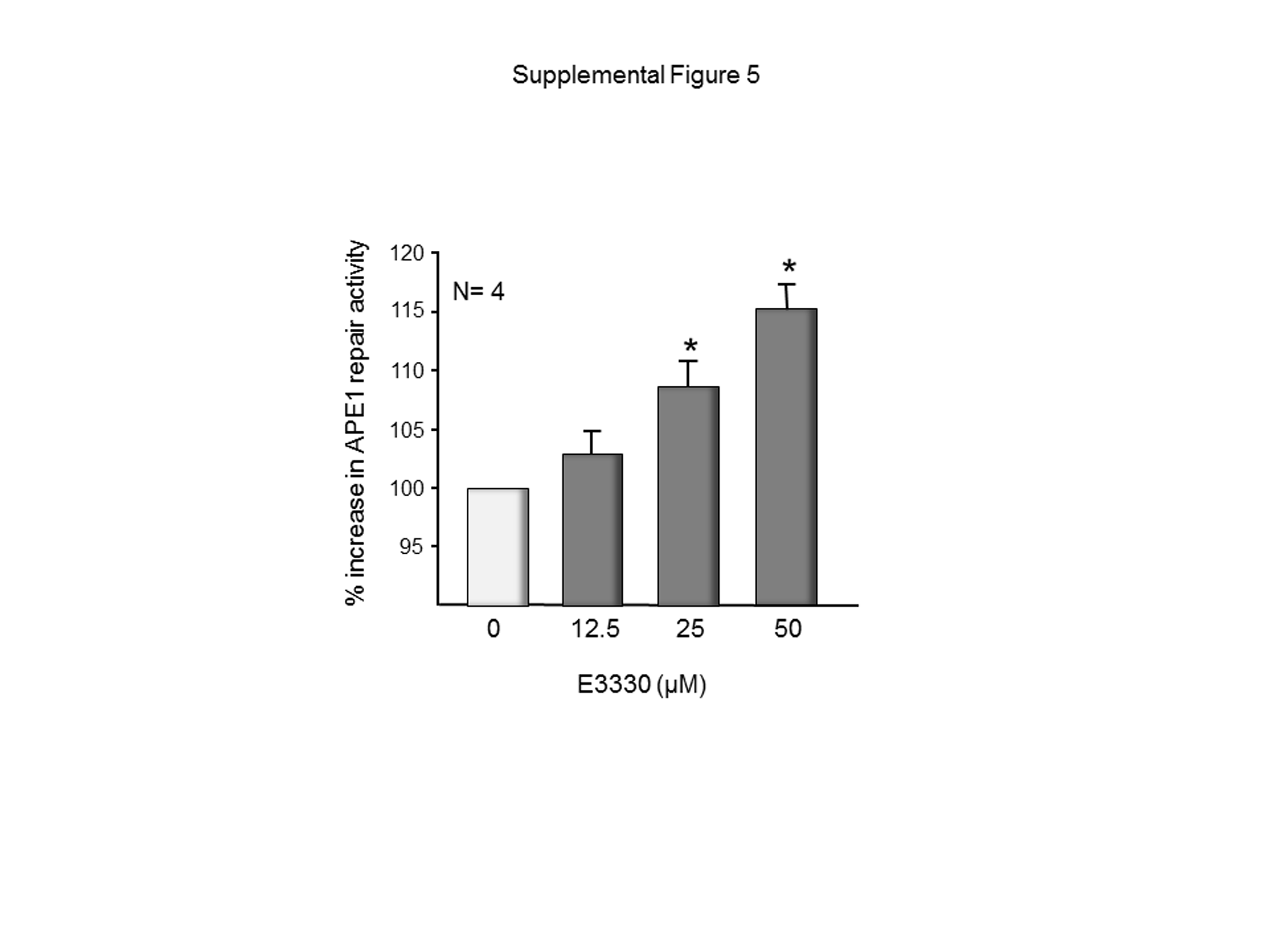

Supplement: Figure S5 — Treating sensory neuronal cultures with E3330 significantly increases APE1 endonuclease activity. Each column is the mean ± SEM of the percent increase in APE1 endonuclease activity using the established AP endonuclease assay (see methods). Activity was measured for extracts of cultures exposed to vehicle control or various concentrations of E3330 for 24 hours as indicated. An asterisk indicates a statistically significant difference between cultures treated with vehicle and those treated with E3330 using Student's t-test. (TIF) [file pone.0106485.s005.tif]
